# From policies to promotion: managers’ views on opportunities for alcohol prevention at work

**DOI:** 10.1080/17482631.2026.2680736

**Published:** 2026-05-26

**Authors:** Kristina Sundqvist, Martina Martinez

**Affiliations:** a Department of Psychology, Stockholm University, Stockholm, Sweden; b Department of Psychology, University of Gothenburg, Gothenburg, Sweden

**Keywords:** Alcohol Prevention, management, organizational factors, alcohol, leadership

## Abstract

**Purpose:**

Managers not only influence employees’ health and well-being, but also play a central role in shaping workplace culture and implementing preventive strategies This qualitative study examined managers´ reflections on the possibilities for alcohol prevention in the workplace.

**Methods:**

Interviews with 44 managers were analyzed using thematic analysis and interpreted through Ames's 1992 four-component cultural model for alcohol prevention.

**Results:**

Four themes described managers perceived possibilities for alcohol prevention: *Shaping social activities*, *Shaping Alcohol Norms,* *Organizational Structures,* and *A Health-Promoting Work Environment*. The first theme concerned integrating alcohol regulations into work-related social activities through limiting servings, offering alcohol-free alternatives, and choosing activities not centered around alcohol. The second concerned influencing the social alcohol climate by placing alcohol on the agenda, role modeling, and encouraging dialogue. The third emphasized how policies, control measures, education, and HR support provide a formal prevention framework. The fourth linked alcohol prevention to broader efforts toward health, psychological safety, and sustainable working conditions.

**Conclusions:**

Managers are central in translating organizational prevention strategies into cultural change embedded in everyday workplace practices. Organizational support, including HR, policies, education, and leadership decision, forms the foundation for long-term preventive work. Addressing alcohol use within broader health and well-being efforts may be an under-recognized prevention strategy.

## Introduction

Alcohol use is recognised as one of the ten leading risk factors contributing to the global burden of disease, accounting for over 5% of all deaths worldwide (Griswold et al., 2018; Shield et al., 2020). Consequently, alcohol consumption remains a significant public health concern. Beyond its impact on individuals and society, high-risk alcohol use negatively affects workplaces through workplace accidents and injuries (McNeilly et al., 2010; Pidd et al., 2006; Ramirez et al., 2013; Soares et al., 2018; Webb et al., 1994), absenteeism (Bacharach et al., 2010; Marzan et al., 2023; Parsley et al., 2022; Pidd et al., 2006; Roche et al., 2016; Schou & Moan, 2016), decreased productivity, impaired job performance (Frone & Bamberger, 2024; Sullivan et al., 2019; Tecco et al., 2013; Thørrisen et al., 2019), and harm to colleagues (Moan & Halkjelsvik, 2020). These consequences highlight the importance of employers addressing alcohol-related risks, particularly as the workplace provides a setting for prevention. In many countries, most adults spend a substantial proportion of their time at work, making the workplace a unique environment for implementing intervention strategies including alcohol prevention efforts (Morse et al., 2022).

From a public health perspective, individuals at risk of alcohol-related problems constitute an important target group for preventive interventions. Most alcohol-related problems in the population arise from drinkers with low to moderate risk rather than from those at highest risk, an observation that forms the basis of the so-called “prevention paradox” (Kreitman, 1986). The prevention paradox refers to the phenomenon that most alcohol-related harms in a population arise from individuals with low to moderate levels of consumption, simply because they constitute a much larger proportion of the population than more heavy drinkers. As a consequence of this distribution, preventive measures that target the whole population may have a greater overall impact on reducing harm than interventions that focus only on high-risk individuals. Universal alcohol preventive strategies at work are considered to have potential since employees who consume alcohol without exhibiting alcohol problems represent a significantly larger group and contribute to a greater overall burden of alcohol-related workplace issues compared to the smaller number of individuals with severe alcohol problems (Pidd et al., 2016).

Findings regarding workplace-based interventions such as information, feedback, health promotion workshops and stress management have been mixed (Fellbaum et al., 2023). While some studies report positive outcomes such as reduced alcohol consumption and improved work engagement, (Bennett et al., 2004; Lee et al., 2014; Spicer & Miller, 2016) others indicate limited behavioural changes (Tinghög, 2014). A recent meta-analysis of both universal and selective workplace interventions found that workplace-based alcohol prevention programmes had a statistically significant and beneficial effect on reducing alcohol consumption (Fellbaum et al., 2023). The varied outcomes can be attributed to differences in study samples, intervention strategies, research methods, and overall study quality. Furthermore, implementing interventions and policies in a workplace setting has proven challenging. Previous studies have identified barriers such as perceived lack of skills and knowledge (Nilsen et al., 2011; Thørrisen et al., 2019), lack of time (Babor et al., 2005; Broyles et al., 2012), limited resources (Johnson et al., 2011; Rojatz et al., 2017), lack of communication, and inadequate strategies (Elling et al., 2022).

Rather than implementing alcohol prevention as a standalone initiative, it may be more effective to embed preventive strategies into a broader organisational framework. Organisational factors, such as leadership practices, workplace norms, work environment structures and job demands, may influence employees’ alcohol use, yet their role in workplace alcohol prevention remains insufficiently understood. Although existing research is limited, studies suggest that organisational factors such as leadership quality (Bamberger & Cohen, 2015; Montal-Rosenberg et al., 2023), workplace drinking norms (Barrientos-Gutierrez et al., 2007; Thørrisen et al., 2022), and job demands (Bamberger & Cohen, 2015; Houdmont & Jachens, 2022) can influence employee alcohol consumption. Furthermore, a recent explorative study, found that organisations with a reduction of employees with high-risk alcohol use were characterised by organisational factors such as clear leadership, continuous and deliberate systematic work environment efforts, ongoing conversation (around policy, guidelines, and core values), and a clear focus on prioritising employee health (Sundqvist et al., 2025). This indicates that organisational factors may represent relevant targets for alcohol prevention. Leadership is particularly important, as managers not only influence employees’ health and well-being but also play a central role in shaping workplace culture and implementing preventive strategies. Therefore, gaining insight into managers’ perspectives is essential for understanding how organisational conditions can either enable or hinder alcohol prevention efforts. By focusing on these perspectives, this study contributes to identifying the organisational factors that can support long-term alcohol prevention in working life.

## Aim and research question

This study aimed to explore how managers perceive the possibilities for alcohol prevention in the workplace, particularly in relation to organisational structures and their own role in shaping preventive practices. Specifically, we aimed to explore: What possibilities for workplace alcohol prevention do managers perceive exist?

## Theoretical model

The environmental approach to the research and prevention of workplace alcohol-related problems considers the differences between individual and occupational influences on alcohol use. Ames and Janes 1992 proposed a cultural model with four interacting conceptual areas for research and alcohol prevention in work life: social control (policies, visibility, and mobility), alcohol availability (physical and social), social/cultural norms (alcohol beliefs, traditions, and rituals), and quality of work life (e.g. stress and job satisfaction). Ames and Janes 1992 proposed that aspects of work culture and work environment can contribute to high-risk alcohol use among individuals, potentially leading to significant and costly work-related issues. Studies later supported two components of the model: availability (Ames & Grube, 1999) and social control (Ames et al., 2000). Social and physical accessibility to alcohol was found to be associated with workplace drinking (Ames & Grube, 1999), and alcohol policies and the extent to which policies were enforced predicted drinking norms and alcohol availability at work, which in turn predicted work-related drinking (Ames et al., 2000). Ames's cultural model did not inform the analytic process; rather, after themes were established, this model was applied in the discussion as an interpretive framework to theoretically contextualise the inductively-generated findings.

## Methods

### Participants

Participants for this study were recruited from the KAPRI project (Elling et al., 2023; Martinez et al., 2022) a Swedish workplace alcohol prevention cluster randomised study combining organisational alcohol policy implementation with skills training. The KAPRI project included 11 medium- to large-sized organisations from hospitality, construction, transportation, insurance, brewing, security, as well as health and social care industries. Data was collected using both quantitative survey data and qualitative interview data from managers and HR officers. This study analysed the interview data; however, while using data from the KAPRI project, it represents a separate study with independent research aims.

Managers and HR officers who attended the two skill‑development workshops were informed about the interview study and were asked if they were interested in participating. Those who expressed interest provided their names on a sign‑up sheet and were subsequently contacted by the interviewer (MM) by telephone or email in the order they had enlisted. Participants who did not respond were followed up to three times by telephone and once by email. Telephone interviews were selected since the participants resided in various cities across Sweden and held demanding managerial positions, making in-person interviews logistically challenging. Additionally, data collection occurred before the COVID-19 pandemic, when digital meeting platforms were not yet widely adopted in professional settings. To ensure depth and openness in responses, all interviews were conducted by a clinical psychologist (MM) with extensive experience in qualitative research interviewing, which was expected to facilitate the establishment of trust and allow participants to discuss sensitive workplace topics in a comfortable manner.

In total, 73 managers and HR officers expressed an interest in participating in the study. Of these, 16 could not be reached, and four declined (all four who declined reported that they had changed their workplace). A total of 53 participants were interviewed, of which 44 were managers and were included in this study. The remaining nine participants were HR officers. Only managers were included in this study as it focuses on the managerial perspective, while HR officer interviews are analysed separately in another study. The participants included both male and female managers with varying levels of experience in their current roles, ranging from less than one year to over 15 years. The majority reported having between three and ten years of experience in their current positions. The organisations represented in the study included nine of the eleven organisations from the original study and all work industries from the original study were represented. The size of these organisations also varied, with employee numbers ranging from approximately 150 to more than 13,000.

Among the managers, the most common roles were the head of operations, department, and section. Other positions included Chief Financial Officer, project manager, executive, and production manager. We classified all managers into three levels: first-line, mid-level, and executive-level managers. First‑line managers are the “immediate managers” responsible for guiding and supporting frontline employees in their daily work. They serve as an essential link between the workforce and upper management to ensure smooth communication, operational efficiency, and healthy workplace culture. In this study, manager positions such as “head of unit”, “team manager”, “team leader, and “site manager” were classified as first-line managers. Mid-level managers bridge the gap between senior leadership and first-line management. This role translates strategic goals into operational plans and ensures that teams and departments work to achieve the organisation’s objectives. Mid‑level managers are responsible for coordinating multiple units or functions, supervising first‑line managers, and monitoring performance across areas of responsibility. In this study, managerial positions such as “head of operations”, ”head of department”, “project manager”, and ”production manager” were classified as mid-level managers. Operating at a broad and strategic level, the highest level of managers focuses on strategic direction, defining goals, allocating resources, and ensuring the alignment of all departments with the organisation’s mission and values. Executive‑level managers oversee the overall performance and sustainability of a business, represent the organisation to stakeholders, and drive innovation and growth. In this study, manager positions such as “CEO”, “CFO”, “head of HR” and “head of finance” were classified as executive level managers. Each quotation in the results section is labelled with the respondent’s ID number and management level (first-line, mid-line, or executive). Detailed participant demographics and organisational characteristics are presented in Table I.

**Table I.** Description of participants.

ID	Management level	Years in current position	Industry	Gender
1	First-line	1−5	Construction	Female
2	Executive	1−5	Hospitality	Female
3	Middle	1−5	Security	Male
4	First line	6-10	Hospitality	Female
5	Executive	<1	Construction	Male
6	Executive	10+	Brewery	Male
7	Mid-level	6−10	Security	Male
8	Mid-level	6−10	Construction	Male
9	Mid-level	1−5	Security	Male
10	Executive	<1	Construction	Female
11	First line	10 years	Health and social care	Female
12	Mid-level	6−10	Brewery	Man
13	Mid-level	6−10	Brewery	Man
14	Mid-level	1−5	Hospitality	Male
15	First-line	6−10	Brewery	Male
16	Mid-level	1−5	Construction	Male
17	Mid-level	1−5	Security	Male
18	First line	1−5	Hospitality	Female
19	Executive	6−10	Hospitality	Female
20	First-line	1−5	Transportation	Male
21	Mid-level	1−5	Construction	Female
22	Executive	6−10	Health and social care	Male
23	First-line	6−10	Health and social care	Female
24	First-line	1−5	Brewery	Male
25	Mid-level	1−5	Security	Male
26	Mid-level	6−10	Security	Male
27	Mid-level	1−5	Security	Female
28	Mid-level	10+	Security	Male
29	First-line	1−5	Brewery	Male
30	Mid-level	1−5	Security	Male
31	Mid-level	<1	Brewery	Female
32	Mid-level	1−5	Health and social care	Female
33	Mid-level	6−10	Security	Male
34	Mid-level	6−10	Security	Male
35	Mid-level	1−5	Security	Female
36	First-line	10+	Insurance	Female
37	Executive	1−5	Insurance	Female
38	Mid-level	1−5	Security	Male
39	Mid-level	<1	Security	Female
40	Mid-level	1−5	Transportation	Female
41	First-line	1−5	Transportation	Male
42	Executive	6−10	Security	Male
43	Mid-level	1−5	Security	Male
44	Mid-level	1−5	Security	Male

### Data collection

Semi-structured interviews were conducted with the 44 managers between September and December 2019. The interview guide was primarily developed by the second author (MM), a licensed psychologist with clinical experience in interviewing and treating individuals with substance use disorders. Input from the other author and the project team resulted in minor revisions; thereafter, the final version of the interview guide was approved by all project members. The interview guide was pilot-tested on two managers prior to finalisation, but no further revisions were considered necessary.

The interview guide consisted of open-ended questions designed to explore the participants’ perspectives and experiences. These questions aimed to explore their general understanding of the issues and phenomena that were investigated, as well as their personal experiences in their professional roles. An example of interview questions that all participants were asked included: “At what stage do you think that you, as a manager, have a responsibility to intervene if you suspect that one of your employees has an alcohol problem?”, “What is the culture regarding alcohol and alcohol-related activities at your workplace?” and “Do you think rules and attitudes among managers toward alcohol can influence employees’ behaviour or attitudes toward alcohol?”. Follow-up questions were utilised to clarify and elaborate on the participants' responses and varied in number and depth, depending on the participants’ initial answers.

Interviews ranged in duration from 25 to 90 minutes, with most lasting approximately 40 minutes. introduction, all participants were provided with information about procedures for ensuring confidentiality as well as being informed of their right to discontinue the interview at any point without having to give the interviewer any explanation.

Each interview was audio-recorded and subsequently transcribed verbatim by a professional transcriber. The interview guide and process were structured in accordance with the methodological principles and recommendations outlined by Braun and Clarke (2022b).

The study was reviewed and approved by the Ethical Review Board of Stockholm Region (no. 2018/634–31/5), and all participants provided written informed consent.

### Analysis

The analysis was conducted by the first author following Braun and Clarke (2022a) six-phase approach to reflexive thematic analysis. Initially, all the interviews were listened to in full to gain an overall sense of the material. This was followed by a thorough reading of the transcribed interviews. In the next phase, all transcripts were coded systematically. In the third phase, the generated codes were grouped into preliminary categories based on the patterns of meaning across the dataset. These categories formed the basis for the development of the initial themes in phase four. In the final phase, themes were reviewed, refined, and named to best capture the underlying ideas expressed in the material. Throughout the process, the analysis was guided by a reflexive approach, meaning that the researcher actively engaged with the data, making interpretative decisions, rather than aiming for objective or replicable coding. The researcher’s own perspectives and contextual understanding were seen as tools for meaning-making rather than sources of bias to eliminate. The researcher (KS) conducting the analysis is a licensed psychologist and has previous experience working with employees with alcohol problems, as well as a (research) interest in the possibility of using the workplace as an arena for alcohol prevention. The analysis was conducted inductively, meaning that themes were generated from the data rather than from a pre‑existing framework. Ames and Janes (1992) cultural model was therefore not used to guide coding or theme development but was introduced later in the discussion to contextualise the four themes.

## Results

The analysis highlights how managers perceive and describe preventive approaches to alcohol consumption in the workplace. Their accounts reflect their perceptions regarding alcohol prevention, including the use of formal organisational structures, shaping social activities, and open communication about alcohol. Some managers also connect alcohol prevention with broader efforts to promote employee well-being. Some managers perceived that addressing alcohol in the workplace was difficult and felt out of place unless there were visible signs or specific concerns. These perspectives are presented in four themes (see Table II), each capturing a different dimension of how managers perceive the possibilities for organisations to include alcohol preventive measures. Each quote is labelled with the respondent’s ID number and management level (first-line, mid-line, or executive).

**Table II.** Summary of results, themes, subthemes, quotes and a description of themes.

Themes	Subthemes	Quote	Description
Shaping Social Activities		“…We go bowling, we do go-karts, we play Fort Boyard-style games. [...] We try to do things that don’t involve alcohol. [...] Basically, we focus on doing activities.”	Strategies to limit the amount of alcohol served, ensure appealing alcohol-free alternatives, and select activities that do not centre around alcohol consumption.
Shaping Alcohol Norms	*Placing alcohol on the agenda*	“I usually bring up things like policies—hygiene, alcohol and drugs, you name it—regularly at our monthly meetings. Just to keep everyone up to date.”	Influencing the alcohol climate by talking about the issue more often, by the way they talk about it, and through their behaviour related to alcohol.
	*Being a role model*	“Even if it’s just a small dinner with your immediate team, the manager who’s present kind of sets the tone for what’s okay.”	
Organisational Structures	*Formal Structures: Policies, Rules, and Responsibilities*	“A policy is a safety net and something to lean on… It’s the source for why I do certain things.”	Formal approaches to alcohol-related issues, including policies, HR responsibility, alcohol and drug testing, and education.
	*Control Measures in Practice*	“We have alcohol locks in every vehicle. We extract logs and check that no one has tried to blow while under the influence.”	
	*Knowledge and a common ground*	“We have recurring training. That’s something that’s really important, and we focus quite a lot on that. Alcohol is part of our training days.”	
Health-Promoting Work Environment		“You create a culture where people feel safe… That’s where I think you need to start—talking about health, how people are doing, stress… Then you can get the conversation going.”	Focus on the work environment and employee well-being in general, rather than on alcohol specifically.

### Theme 1: Shaping social activities

This theme encompasses efforts to create social events where participation is not dependent on alcohol consumption. It includes strategies to limit the amount of alcohol served, ensure appealing alcohol-free beverage alternatives, and select activities that do not centre around alcohol consumption.

The most common idea among managers regarding alcohol prevention in the workplace was to limit the amount of alcohol served during work-related events. Almost all participants stated that employees were offered no more than two to three alcoholic beverages, if any, during work dinners or celebrations. “We’re very restrictive when it comes to those things. For example, at staff parties, you might get two drink tickets for Christmas dinners. That’s it” (IP 12, mid-level).

Some managers also emphasised that strong spirits were never served, with alcohol offerings limited to beer or wine. A few managers further noted that their organisations had reduced overall spending on representation and formal events involving alcohol as part of a more restrictive approach.

In addition, some managers emphasised the importance of offering appealing non-alcoholic alternatives. This was seen as a way to make alcohol-free choices more attractive and socially acceptable during workplace events. “There should always be non-alcoholic alternatives. That’s important. There should be at least as many of those, and it should be completely okay to choose alcohol-free options… That is probably the most important thing” (IP15, first-line).

Some managers described a preventive approach that involved moving away from traditional social events in which alcohol is typically served. Instead, they suggested alternative ways of celebrating or gathering that did not involve alcohol at all. One example mentioned by several participants was celebrating with cake instead of champagne or other alcoholic beverages. Others suggested choosing activities not associated with drinking, such as going to places where alcohol is not served.

We try to come up with more things to do with our employees when we arrange something… We go bowling, we do go-karts, we play Fort Boyard-style games. [...] We try to do things that don’t involve alcohol. [...] Basically, we focus on doing activities (IP 25, mid-level).

In summary, this theme highlights attempts at workplaces to reduce alcohol-related risks for employees by limiting the alcohol served in work-related contexts, by normalising alcohol-free participation, and by fostering a workplace culture in which activities other than those associated with alcohol consumption are prioritised.

### Theme 2: Shaping alcohol norms

This theme concerns how managers view the possibilities of influencing the alcohol climate by discussing alcohol more frequently, by shaping the way alcohol is communicated, and through their own behaviour related to alcohol.

#### Placing alcohol on the agenda (Talking about it)

##### *At onboarding*.

Several managers emphasised that a key moment for influencing alcohol-related norms and expectations is the onboarding of new employees. They described how alcohol policies and attitudes are typically introduced early in the employment process, sometimes even before a new employee formally begins working. Formal procedures were common, such as distributing and signing policies: “They read through it, they sign it, and it’s filed with their employment documents. So they can’t come later and say they haven’t seen it” (IP 41, first-line). One manager explained that the onboarding process is an opportunity to communicate expectations to employees: “We make it clear that we have zero tolerance—that when you’re at work, you’re sober. That’s just how it is. You can’t let it affect your job. It’s part of the expectations we have of our staff” (IP 35, mid-level).

Several managers emphasised the importance of communicating not only the rules but also the underlying values: “This is a workplace issue, especially in the industry I work in. So we bring it up during interviews, and then again during the introductory training. It’s about values, guidelines, policies, systems—everything you need in order to do your job” (IP 42, executive). This quote illustrates how conversations about alcohol go beyond rules but also explains the reasons for preventive measures; whether related to workplace safety or solidarity with colleagues who may struggle with alcohol problems. In this way, managers aim not only to ensure compliance but also to shape employees’ attitudes toward alcohol and socialise them into workplace culture.

The onboarding process was considered important for making alcohol-related rules and values explicit. Whether it involves having employees sign an alcohol and drug policy or more informally making clear that the workplace has shared norms around alcohol, such conversations were considered beneficial in shaping the broader workplace alcohol climate.

##### *In connection with activities*.

The managers also described that integrating discussions about alcohol into various work activities enabled placing alcohol on the agenda. These activities, which ranged from staff parties to random breathalyser cheques, were regarded as natural and recurring opportunities to raise the subject and remind employees of the organisation’s expectations about alcohol.

Several managers talked about having these conversations in connection with events where alcohol is present. One manager reflected on how staff parties are used as a platform to discuss company policies and set boundaries: ”…we bring it up at every staff party to emphasise why the limit is two beers. It's not stinginess—it’s about something bigger than that (IP 19, executive).”

Several managers described how testing occasions could be used to create awareness of alcohol use in addition to working as enforcement. As one manager explained, “We do six or eight breathalyser rounds per year, a bit irregularly… And then, of course, people talk about alcohol when you are doing it” (IP 29, first-line). This suggests that control situations are used as a natural opportunity to raise the topic of alcohol in a practical and non-confrontational manner, making it easier for managers and employees to talk about alcohol. Such situations can create moments where norms and values around alcohol can be addressed naturally and, as a result, gradually reshaped within the organisation.

Creative approaches, like involving credible outside speakers or placing self-assessment forms in common areas, were also used as a starting point for conversations about alcohol and to encourage reflection on one’s own consumption: “We put the forms out by the coffee tables, and yes, people joked a bit, but many filled them out and talked about it. Of course, they did—it was there” (IP 38, mid-level).

##### Continuously.

Several respondents proposed addressing alcohol issues continuously and proactively in the workplace. Rather than waiting for problems to arise, it is about building a culture of awareness and prevention, where the topic is regularly brought up in staff meetings, check-ins, or other workplace routines. Managers described how alcohol prevention can be integrated into everyday routines by regularly addressing related topics in internal newsletters and meetings. By asking team leaders about staff well-being, they aim to catch early signs of employee health issues for which alcohol might be an underlying factor. “I usually bring up things like policies—hygiene, alcohol and drugs, you name it—regularly at our monthly meetings. Just to keep everyone up to date” (IP 29, first-line). Even respondents who did not work in this way saw the preventive potential of including alcohol as a topic in everyday practice. As stated by one manager:

It’s better if something can be prevented before becoming a problem. So, it’s important, and maybe something that should be discussed at staff meetings… We have several staff meetings a year—that could be a point on the agenda. Talk about drugs and alcohol, maybe in a more casual way, just to get people to think. (IP 30, mid-level).

However, establishing a regular routine to discuss alcohol in the workplace can be difficult, particularly when the issue is not perceived as urgent. In contrast, some managers expressed that the organisation typically addressed the topic when something had gone wrong and not before. This includes alcohol-related incidents or accidents: “It always ends up being reactive. You act after something has happened. However, there needs to be continuity—that it’s talked about regularly, not once every ten years” (IP33, mid-level). Some managers expressed that alcohol was considered a private matter or a taboo topic, making it difficult to address. One manager compared it to other sensitive issues, explaining that it is not something you bring up unless there is a reason to:

Yeah, well, no. For me, it’s like this. Bringing something up in a group where there’s generally no concern or thought about it at all makes it very difficult to approach. Because it feels like if I were to start talking about eating disorders with my staff, just because it might be something dangerous for some people, that they could develop anorexia? No, I don’t do that. It’s like alcohol—it’s something you address in the moment, when you suspect or notice something. But it’s very difficult to talk about this preventively before. (IP 32, mid-level)

Yet others saw it as a leadership responsibility to raise awareness continuously, beyond just having a written policy: “It’s not just about having a policy on paper. It is about raising awareness and keeping the issue alive for some time. That has more effect” (IP 38, mid-level).

Several managers expressed that creating a safe environment in which employees feel comfortable talking to each other has the potential to help prevent alcohol-related issues and that talking about alcohol can also reduce the stigma surrounding the topic, making it less taboo. ”You can have forums where you just talk about it—where you try not to make it a taboo, but instead talk about it and bring up the topic, even if there's no problem” (IP 11, first-line). Another manager stated, ‘No, I’d say it’s really about being open—having a workplace or company culture where you talk about these things and don’t make them a taboo” (IP 42, executive).

#### Being a role model

A recurring theme in the interviews was how leadership behaviour influences workplace alcohol culture. Even small actions, such as what a manager orders at a dinner, can influence what employees perceive as acceptable. Many respondents emphasised that leaders “set the tone,” whether they intend to or not. Their choices become informal rules: “Even if it’s just a small dinner with your immediate team, the manager who’s present kind of sets the tone for what’s okay” (IP 1, first-line).

A manager’s behaviour was considered to affect both how much people drank and whether they felt pressured to conform. The managers expressed that leaders who drank heavily or encouraged others to drink, sometimes unintentionally, were considered to have a poor influence on their co-workers. In contrast, respondents expressed that managers with restrictive alcohol-related habits could have a positive impact on their co-workers’ drinking habits. “If I post pictures from a management meeting where I’m drinking heavily, of course that affects the group. When I stay sober, it tends to dampen the party mood. But maybe that’s for the best” (IP 38, mid-level).

The expectation that leaders would act as role models emerged repeatedly. This is not only about what they say. Rather, it is about the consistency between words and actions: “If you want others in the organisation to take these issues seriously, then it’s really important to lead by example. If we want middle managers to act, they need strong role models from the top” (IP 10, executive).

In summary, this theme emphasises how clear communication about alcohol rules and values, together with acting as a role model, may contribute to a workplace culture in which conversations about alcohol are more normalised and perceived as less stigmatising.

### Theme 3: Organisational Structures

This theme captures the formal and systematic approaches used by organisations to manage alcohol-related aspects in the workplace. This includes, among others, having established policies and clear rules, an HR department responsible for alcohol topics, and the implementation of random alcohol and drug testing. These structures reflect the organisation's responsibility to proactively monitor and regulate alcohol use and ensure that prevention is embedded in everyday operations.

#### Formal structures: policies, rules, and responsibilities

One of the most consistent organisational structures referenced by managers was the presence of a formal written alcohol policy. These policies were generally described as foundational documents that establish clear expectations about alcohol use and function as a guide for both preventative efforts and acute interventions.

Several managers highlighted that having a written policy offers both practical support and psychological reassurance. As one interviewee stated, “A policy is a safety net and something to lean on… It’s the source for why I do certain things” (IP 39, mid-level). Another respondent emphasised the stabilising role that policies play, especially for less experienced leaders: “It’s a support, absolutely… especially when you're new and expected to handle things, it’s comforting that there are rules and instructions to lean on” (IP 9, mid-level).

As described in Theme 2, respondents frequently noted that signing the policy was part of the hiring process. Information about alcohol and alcohol guidelines tended to be presented to employees while they were simultaneously being briefed with other forms of introductory information, which ensured that all new employees were made aware of the organisation’s stance on alcohol. However, managers also recognised that the real challenge lay not in having the policy but in keeping it active and relevant. As one noted, “We hand it out during onboarding… but then it gathers dust until a problem arises” (IP 23, first-line).

Despite these limitations, many leaders still viewed the existence of alcohol policy as essential, both symbolically and practically. It was considered to signal that the organisation takes alcohol-related questions seriously and that the policy may function as a guiding tool in daily work; however, its significance is most evident when sensitive alcohol-related situations occur. As one manager summed it up: “It shows that we take these things seriously… even if I can't quote it word for word, I know it's something we will act on” (IP 19, executive).

Finally, several managers emphasised that HR plays a central and guiding role in alcohol prevention within their organisations. HR departments are seen as the main drivers of policy implementation and support for line managers. As one interviewee described: “We have a personnel department that focuses on these things, supports us, and provides good conditions. But generally, in the company, they are the ones who lead this preventive work“ (IP 12, mid-level).

The reliance on HR for both guidance and reassurance was also emphasised when handling cases where an employee appeared to be struggling with an alcohol problem. “I would go to our HR“ said one manager (IP 18, first-line), when asked how they would act upon concerns about an employee’s alcohol use. Another emphasised the importance of HR as a resource: “It’s hard to act alone, I would quickly seek that support“ (IP 21, mid-level). Although prevention was the focus of this study, many participants still referred to specific situations at work in which they had encountered employees with alcohol issues. Having HR as a structured support system appeared to reduce uncertainty and give managers confidence in responding to situations.

#### Control measures in practice

A recurring aspect of organisational structures for alcohol prevention in the workplace was the use of control measures such as random testing and technical interventions like alcohol locks. These were described both as preventive tools and ways to uphold clear boundaries around sobriety at work. Many managers emphasised the routine implementation of breathalyser tests. These were often framed as being part of everyday procedures rather than as exceptional interventions. One manager explained: “We have a breathalyser here, it’s part of the job that we do breathe tests now and then. These tests can happen at night, during weekends, weekdays, everything“ (IP 29, first-line). Control systems also included technical aids, such as alcohol interlocks in vehicles, which were highlighted as both safety measures and preventive tools in sectors with frequent driving. Although some interviewees acknowledged that these measures may be seen as excessive or unnecessary, especially when no one “gets caught“, they were still seen as contributing to a more open dialogue about alcohol in the workplace.

#### Knowledge and a common ground

A frequent aspect identified in the interviews was the role of education in supporting alcohol prevention efforts at the organisational level. Several managers emphasised that structured and repeated training contributed not only to knowledge dissemination but also to creating space for dialogue and reflection. One manager explained: “…we talk more about it now than we did two years ago, definitely. And this training is a part of that. After that we’ve definitely started to talk a lot more about it in different contexts“ (IP 1, first-line).

Ongoing training and recurring educational efforts were seen by some as key components of alcohol prevention. These opportunities also allowed for experience-sharing among colleagues, which was perceived as valuable in translating theoretical knowledge into practical insights. As one manager described: “We had a recurring training. That’s something that’s really important, and we focus quite a lot on that. Alcohol is part of our training days“ (IP12, mid-level).

Several managers pointed to the importance of gaining practical tools and confidence in addressing sensitive situations. One participant reflected: “Give me more information and give me knowledge and I will use it. That’s really what it’s about“ (IP 26, mid-level). Another manager emphasised that education was important not only for strengthening individual knowledge and skills, but also to ensure a shared and consistent understanding across the organisation: “Before, there was little, if any, information or lectures. It was more like, ‘here’s the policy, follow it.’ But now you’d want everyone to get the same education or lecture, so there’s a common baseline“ (IP 43, mid-level). When everyone in the organisation receives the same education about alcohol, the rules and expectations become clearer and more likely to be interpreted in the same way.

In summary, managers described organisational structures as central for enabling alcohol prevention at work. Policies and clear rules provided guidance in sensitive situations, HR personnel offered essential support, control measures reinforced expectations around sobriety, and training contributed to shared knowledge and confidence in addressing alcohol‑related issues.

### Theme 4: A health-promoting work environment

This theme reflects the efforts within organisations to create a safe, supportive workplace that promotes long‑term employee health and well‑being. It encompasses both the physical and psychosocial aspects of the workplace, aiming to reduce stress, promote mental and physical health, and foster a climate of trust and inclusion. Initiatives within this theme include open communication, psychological safety, as well as efforts to ensure that work practices are designed to be sustainable over the long term, without risking employee health or well-being. The focus is on preventive and promotive strategies that support employees not only in avoiding harm, but also in thriving at work. The theme reflects managers’ views on general workplace well‑being as an indirect pathway to preventing alcohol-related problems, rather than alcohol-specific measures. It should be noted that this perspective on alcohol prevention was the least common among the managers. Their views ranged from those not seeing the opportunity at all—“I cannot see the connection between wellness or this being healthy and alcohol at all“ (IP 7, mid-level)—to those acknowledging it but considering it difficult. As one participant responded to the question of whether alcohol appears as a health issue:“We would need to do that, but again, it becomes so… It always turns into such a taboo around the whole thing” (IP 23, first-line).

Although not extensively discussed, several managers emphasised the importance of creating a healthy and supportive work environment. These efforts were generally framed within broader strategies to ensure employee well-being rather than being explicitly aimed at alcohol use. Both physical and psychosocial work environment measures were mentioned. One manager pointed out that organisational changes, such as shifts in staffing or schedules, can have consequences for employees' overall well-being, which in turn may influence their coping mechanisms:

If someone ends up in a situation with poorer conditions, socially or at work, that could be a contributing factor to different kinds of problems… Some people might find it easier to turn to alcohol as a way to escape or temporarily feel better (IP 12, mid-level).

Another emphasised the role of basic working conditions and psychosocial support, explaining that attention to daily needs and mental well-being can help prevent alcohol use: “You have to be able to heat your lunch box. You have to be able to use the toilet… If you provide people with those basics, I believe that affects everything else“ (35, mid-level). This manager also stressed the importance of open dialogue and workplace culture, saying: “You create a culture where people feel safe… That’s where I think you need to start—talking about health, how people are doing, stress… Then you can get the conversation going“ (IP 35, mid-level).

In a similar manner, another respondent described preventive work as part of a broader responsibility to ensure balanced working conditions: “Somewhere I believe in that balance in life… that the employer provides good conditions” (IP 17, mid-level).

Only a few managers mentioned the use of employee or health surveys as part of alcohol prevention efforts. When such measures were discussed, they were often described as general tools for monitoring employee well-being, rather than being specifically focused on alcohol.

One manager explained that survey questions include ”…drinking habits. How often do you drink? And in what quantities?” noting that the questions are “very general” and are part of broader employee surveys used across Sweden (IP 12, mid-level). Another noted that their organisation includes alcohol-related questions in employee surveys “like how many glasses you usually drink per week: is it once a week, once a month, or more often?” (IP 31, mid-level).

Overall, while surveys and check-ups were mentioned, their connection to alcohol prevention appeared limited and not consistently implemented. However, a few managers viewed alcohol as part of a general health perspective.

You don't have to talk about alcohol in those specific terms—you can talk about health. And when you talk about health, we encourage people to think about their well-being, and the company's perspective is that we want people to feel good and eat well, and then you can include it in that context. It’s like with any kind of training. You can pose a question to your staff: What do you consider to be use? Is it when I drink every day, or only on weekends, and so on? That way, you can create a dialogue and discussion, and make people more aware of it. And that’s something we’ve done (IP 24, first-line).

Most of those who described alcohol as *not* being part of the health perspective still regarded it as being desirable. When asked whether alcohol was considered part of a health context, one participant said:

No. That’s where we are again. That’s exactly what it doesn’t do. It doesn’t—not on that level. Or, well, it’s barely even talked about in these contexts. We really should talk about it, but again, it becomes so… there’s such a taboo around it all the time (IP 23, first-line).

In sum, these accounts suggest that while not always labelled as alcohol prevention, promoting a healthy and responsible work environment was seen by some managers as indirectly supporting such efforts.

### Synthesis of results

**Figure 1.**
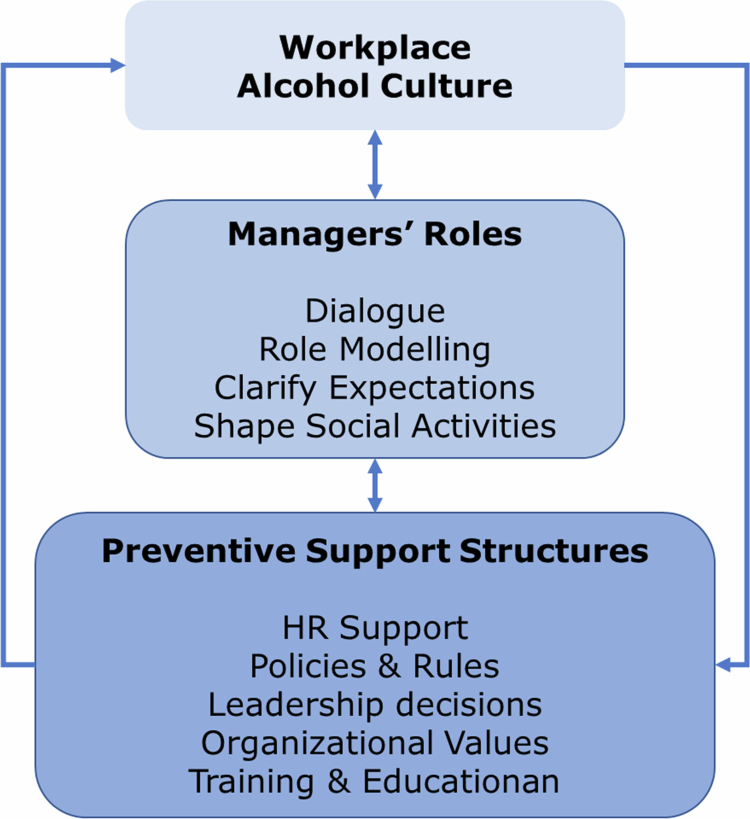
An illustration of the reciprocal relationship between managers and organisational structures in enabling change in the workplace alcohol culture.

The themes illustrate how workplace alcohol prevention depend on the interaction between managers’ roles and organisational support structures. Managers are positioned as key actors who shape everyday practices and expectations related to alcohol. Their behaviour and communication signal what is considered acceptable, thereby contributing to the development of shared norms around alcohol use in work-related contexts. At the same time, managers’ actions are supported and enabled by organisational structures and employee-centred values. These structures provide legitimacy, guidance, and practical tools to support managers in shaping of the workplace alcohol culture. Figure 1 illustrates the reciprocal relationship between managers and organisational structures in enabling changes of the alcohol culture.

## Discussion

This study examined managers ´ experiences and perspectives on the possibilities for alcohol prevention in the workplace. The themes generated through inductive thematic analysis: (1) *Shaping social activities*, (2) *Shaping Alcohol Norms,* (3) *Organisational Structures,* and (4) *A Health-Promoting Work Environment*, are here discussed from the perspective of Ames and Janes 1992 cultural model. Ames’s model identifies four interrelated conceptual areas that influence alcohol-related behaviours in the workplace: *social control, alcohol availability, social and cultural norms*, and *quality of work life*. Using this cultural model as a basis for our discussion allowed us to contextualise our results from a theoretical perspective.

### Social control

Managers highlighted various mechanisms as key components of alcohol prevention, that aligned with Ames’s framework of social control in the workplace. They emphasised the role of formal alcohol and drug policies in establishing clear organisational expectations and norms regarding alcohol use. Managers’ views on using alcohol policies in organisations align with previous research showing that workplace social control in the form of alcohol and drug policies is associated with reduced alcohol consumption among employees (Alfred et al., 2021; Pidd et al., 2016).

Managers also described regular alcohol and drug testing and interlocks in company vehicles as routine practices that promote responsibility and the vision that sobriety is expected. Beyond ensuring safety, these tools may signal a workplace culture of responsibility to employees. However, systematic reviews indicate that workplace alcohol testing has limited impact (Cashman et al., 2009; Pidd & Roche, 2014). Because much of the available evidence is of low quality, the effectiveness of such measures in reducing substance use or in improving safety remains uncertain (Pidd & Roche, 2014). Nevertheless, in this study, the managers conveyed that such control measures not only enforce rules but also create opportunities for informal conversations about alcohol use, contributing to the normalisation of the topic. This reflects how the conceptual areas proposed by Ames are interconnected and mutually influence each other; in this case, social control measures can be used to shape social and cultural norms around alcohol.

### Alcohol availability

By limiting the amount of alcohol served and by offering more non-alcoholic alternatives, managers directly address one of Ames’s key risk factors; availability. Their focus on availability reflects an understanding that access to alcohol may influence employees’ drinking behaviour and workplace alcohol culture. Limiting the availability of alcoholic beverages and increasing non-alcoholic options can help normalise the choice to abstain from alcohol. Promoting non-alcoholic beverages in the workplace is also a way to enhance inclusion, as some employees prefer to not drink. Research has shown that lower social and physical accessibility to alcohol reduces undesirable drinking that occur just before coming to work and during work hours (Ames & Grube, 1999), supporting managers’ perceptions that limiting alcohol availability may function as a preventive strategy. This may be beneficial because alcohol consumption is associated with health risks, even at low levels (Shield et al., 2020).

Finally, although managers frequently discussed prevention in terms of limiting alcohol availability at workplace events, this focus implicitly assumes that alcohol is an expected part of workplace socialising. Such assumptions have been problematized in recent literature, which highlights how alcohol‑centred events may inadvertently exclude employees who abstain for reasons related to health, religion, recovery, or personal preference (Ellythy et al., 2024). From this perspective, alcohol‑free events or policies restricting the use of company funds for alcohol may represent additional strategies for fostering inclusion and reducing alcohol‑related risks. These approaches extend beyond regulating availability by reconsidering whether alcohol needs to be integrated into workplace gatherings at all; an idea that a few managers implicitly raised when reflecting on alternatives to serving alcohol at workplace events. Most state-owned companies in Sweden, for example, do not allow company funds to be used for the purchase of alcoholic beverages.

### Social and cultural norms

Managers described how their organisations intentionally reshape workplace social events to shift norms around alcohol, actively challenging established associations between alcohol and celebrations, rewards, or employee bonding. This is supported by Ames’s claim that workplace culture has the potential to either reinforce or reduce alcohol use. The managers in our study seemed aware of how social events including alcohol, such as staff parties and formal dinners, could make abstainers feel excluded. By promoting practices that support inclusive participation regardless of alcohol use, managers can help contribute to reshaping what is socially acceptable and expected. Such restrictions align with research showing that when alcohol is served more frequently at work, employees are more likely to perceive higher levels of drinking as risk-free; an attitude that may also contribute to increased alcohol consumption (Sundqvist et al., 2025). Furthermore, in accordance with the prevention paradox, the largest share of alcohol‑related harm arises from the much broader group of low‑ to moderate‑consumers rather than from those typically perceived as high‑risk consumers. Consequently, measures that limit availability and promote alcohol‑free options may help reduce overall alcohol consumption and contribute to a reduction in alcohol‑related harm.

In addition, managers described how they used naturally occurring situations to initiate conversations about alcohol. The idea that discussing alcohol is “not taboo” may foster an environment in which employees feel safer discussing concerns and where prevention is integrated into everyday culture rather than being reactive or only brought up when necessary.

Managers also described their role in influencing cultural norms through their behaviour. Choices and actions, such as if and how much they drink during social events, can be interpreted as informal cues that contribute to shaping group expectations.

The role of managerial behaviour, often intentional and sometimes unintentional, in influencing organisational cultural norms is supported by research (Klein et al., 2013). Furthermore, norms have been identified as some of the strongest predictors of employees’ alcohol consumption (Barrientos-Gutierrez et al., 2007). For example, a Norwegian study found that liberal workplace drinking norms were strongly associated with higher alcohol use, even after controlling for other workplace and individual factors, concluding that norms were the most influential workplace factor (Thørrisen et al., 2022). However, strategies for reshaping alcohol-related norms remain unexplored and need to be investigated further.

### Quality of worklife

Only a few managers explicitly connected efforts to improve the quality of the work environment with alcohol prevention. While many emphasised the importance of creating a safe and supportive, workplaces that promotes employee well-being, this was generally framed as a broader health or organisational responsibility rather than being specifically associated with alcohol prevention.

Managers highlighted physical and psychosocial factors, such as workload, stress management, and open communication, as crucial for employee health and well-being, yet few linked these directly to alcohol-related risks. In terms of alcohol consumption, some managers found it difficult to bring up alcohol in such contexts. The difficulties associated with discussing alcohol with a colleague are supported by previous research, which demonstrated that employees found it considerably easier to talk to their co-workers about stress-related issues, compared to discussing alcohol (Thoreson et al., 1986). Although conventional tools, such as employee health surveys, were mentioned by some managers, they were rarely used for the purpose of alcohol prevention, as alcohol was seldom framed as part of overall health and well-being. This indicates that the connection between quality of work life and alcohol prevention may be unrecognised or implicit, rather than conscious.

Nevertheless, Ames’s model suggests that poor quality of work life, characterised by stress, low job satisfaction, and unsupportive conditions, can increase high-risk alcohol use. From this perspective, managers’ focus on improving overall work conditions, even when not directly associated with alcohol, may still yield preventive benefits. This interpretation is supported by earlier research showing that employees who participated in a stress management programme reported significant reductions in alcohol consumption, even in the absence of substance-use-specific interventions (Cook et al., 2003). Similarly, studies of interventions aimed at strengthening team communication, support, and stress management have demonstrated a reduction in drinking, further implying the potential benefits of preventive workplace well-being initiatives (Bennett et al., 2004).

The limited extent to which managers viewed alcohol as a physical health issue is noteworthy, given the well‑established evidence linking alcohol use, even at low levels, to increased risks of cancer, chronic disease, and injury (Shield et al., 2020). This raises questions about how alcohol could be more explicitly integrated into workplace health and well‑being efforts. Previous research demonstrates that health‑promotion approaches, such as lifestyle campaigns, stress‑management programmes, and web‑based personalised feedback, can reduce risky drinking as part of broader health initiatives (Ames & Bennett, 2011). Several managers noted that their organisations routinely include alcohol‑related questions in employee surveys, yet these data are seldom used proactively for preventive purposes. Future interventions could draw on such tools to provide individualised feedback, support early identification, or link alcohol to health promotion efforts. Exploring how alcohol prevention can be framed as part of occupational health, rather than solely as a behavioural or cultural issue, may broaden preventive possibilities and align workplace strategies more closely with contemporary public health evidence.

In summary, the findings show that managers’ perceptions of prevention possibilities correspond to all four Ames’s conceptual areas. Availability and social control were addressed more directly and consciously, while the influence on social norms was often more indirect, for instance, by raising the subject more frequently. Some measures also appeared to serve dual functions, depending on how they were implemented. For example, sobriety cheques not only enforced rules, but also opened up conversations about alcohol. In contrast, the link between quality of work life and alcohol prevention was seldom explicit, indicating a considerable scope for further development in this area.

### Limitations and future directions

All managers in this study had previously participated in brief training on alcohol in the workplace, as their organisations were involved in a larger study (Elling et al., 2023; Martinez et al., 2022). Participants also self-selected by signing up for the study, which may indicate a higher level of interest or awareness of the topic compared to managers in other organisations, potentially limiting the transferability of the results. The findings are also context-bound to Sweden and to medium- and large-sized organisations (≥150 employees), particularly those with established HR departments, formal policy frameworks and hierarchical management structures, making generalisability to smaller organisations limited. Additionally, transferability to countries with different alcohol norms, workplace cultures, or legal frameworks regarding employee health should be considered carefully, since responsibilities and possibilities for prevention may vary substantially across organisational and cultural contexts.

In addition, the use of telephone interviews, while practical given the geographical spread of participants, limited the possibility of observing non-verbal cues that could have added depth and context to the data. However, the telephone format may also have reduced social desirability bias by offering participants a greater sense of anonymity when discussing a sensitive topic.

Finally, the analysis followed reflexive thematic analysis (RTA) led by the first author. While appropriate for exploring under-researched phenomena, the absence of additional strategies such as analyst triangulation or member checking may limit the credibility of the findings.

Future research should examine how alcohol prevention can be integrated more clearly into existing organisational structures. Several managers noted that alcohol is seldom addressed as a health issue, despite well‑established links to physical health risks even at low levels of consumption. Exploring approaches that incorporate alcohol into routine health promotion efforts or existing work environment processes, for example through employee surveys or risk assessments that are already in place, may offer new opportunities for early identification and more systematic preventive work. Such integration could broaden the range of feasible workplace interventions and strengthen the long‑term sustainability of prevention efforts.

## Conclusion

This study indicates that managers play an important role in transforming organisational alcohol prevention strategies into genuine cultural change and fostering workplace practices that promote consistent and long‑term alcohol prevention. By placing the issue on the agenda, serving as role models, and encouraging open dialogue, managers can influence everyday attitudes and behaviours. However, this work is often considered challenging, particularly when discussions surrounding alcohol are regarded as private or taboo.

For preventive efforts to be effective, success depends not only on committed managers, but also on clear organisational decisions and robust support structures. HR and senior leadership must take active responsibility for establishing frameworks, ensuring follow-up, and maintaining continuity, so that alcohol prevention does not become an isolated responsibility for individual managers.

Finally, alcohol was rarely framed as a health issue, highlighting an overlooked potential to strengthen prevention by integrating alcohol into broader strategies for promoting employee health and well-being. When leadership, HR officials, and managers work together to integrate alcohol-related issues into broader strategies for health, workplace culture, and organisational values, the potential for lasting changes in the alcohol culture of the workplace increases.

## Data Availability

Due to the sensitive nature of the topics discussed and confidentiality agreements with participants, the full interview transcripts cannot be shared publicly. The following materials are available from the corresponding author upon request, subject to the requester obtaining ethical approval: de-identified interview excerpts, the coding framework, and thematic maps.
